# Association between triglyceride glucose combined with body mass index and hypertension in the NHANES 2017 to 2020

**DOI:** 10.1038/s41598-025-93723-w

**Published:** 2025-03-17

**Authors:** Lin Zhao, Liying Zheng, Rumeng Wang, Xiao Gong, Yanyan Wu, Senfu Han, Leshun Liu, Mei Xue

**Affiliations:** 1 Xiyuan Hospital, National Clinical Research Center for Chinese Medicine Cardiology, Academy of Chinese Medical Sciences, Beijing, China; 2 Xiyuan Hospital, China Academy of Chinese Medical Sciences, Beijing, China; 3 Beijing University of Chinese Medicine, Beijing, China

**Keywords:** TyG-BMI, Hypertension, Insulin resistance, NHANES, Cross-sectional study, Risk factors, Cardiovascular diseases

## Abstract

Triglyceride glucose-body mass index (TyG-BMI), as a new surrogate index for evaluating insulin resistance (IR), has rarely been studied with the incidence rate of hypertension (HTN). This study aims to explore the correlation between TyG-BMI and HTN in American adults, to find a cost-effective and convenient marker to guide early prevention of HTN. We performed this cross-sectional study based on the NHANES database from 2017 to 2020. A multivariate logistic regression model was used to analyze the correlation between TyG-BMI and the prevalence of HTN. Additionally, stratified analysis was performed to test the robustness of the results. 3,069 eligible participants were included in our study (48.8% male, mean age 50.0 ± 17.2 years), and multivariate logistic regression analysis showed that TyG-BMI was maintained positively correlated with HTN after fully adjusting covariates. For every 10-unit increase in the TyG-BMI, the risk of HTN increases by 4.3% (95% CI: 1.007–1.08, P = 0.018). When TyG-BMI was represented as a Quartile, the relationship between TyG-BMI and increased risk of HTN remained significant, which were statistically significant for each model. Stratified analyses suggested that the correlation between TyG-BMI and HTN was more pronounced in those aged 60 years or older (P < 0.05). The correlation between TyG-BMI and HTN remained stable in all strata except the age subgroup (P > 0.05). TyG-BMI is significantly associated with HTN among American adults. Early monitoring of TyG-BMI may help to monitor early the risk of hypertension.

**Supplementary Information:**

The online version contains supplementary material available at 10.1038/s41598-025-93723-w.

## Introduction

Hypertension (HTN) is a major cause of the increasing economic burden of disease and mortality globally, with approximately 30% of adults worldwide currently suffering from HTN and 50–60% of adults in pre-hypertension^1,2^. The existence of a large number of pre-hypertension individuals indicates that the incidence of HTN will continue to increase in the future^3^. In addition, during HTN, peripheral arterial pressure increases, heart load increases, and vascular endothelial damage occurs, leading to damage to multiple target organs including the heart, brain, and kidneys. Therefore, prevention and management of HTN is a major challenge in global public health care.

Insulin resistance (IR) is closely related to the occurrence of HTN^4,5^. IR can lead to increased secretion of Ang II (Angiotensin II, Ang II) and aldosterone, excessive activation of the sympathetic nervous system, and promotion of oxidative stress, leading to HTN^6–9^. However, the “gold standard” of assessing IR, the high insulin-normal glucose clamp (HIEC), is an invasive technique that is complex, time-consuming, and expensive, and therefore not suitable for large-scale studies. Homeostasis model assessment (HOMA-IR) is an alternative metric to assess IR and has been shown to strongly correlate with HIEC, but it is also not suitable for large-scale clinical investigations due to the need to assess insulin levels and the high variability of HOMA-IR threshold levels. For the past few years, the triglyceride glucose (TyG) index and related parameters [Triglyceride glucose-Waist Circumference (TyG-WC), TyG-BMI] have become new alternative indicators for evaluating IR, characterized by simplicity, ease of operation, and low-cost, making it suitable for large-scale clinical screening. The existing research has confirmed that TyG is associated with the incidence rate of many diseases in combination with WC, waist hip ratio (WHR) and waist height ratio (WHtR)^10^. Among them, TyG-BMI is most closely related to HOMA-IR^11–15^. IR and obesity are closely related to the risk of hypertension. The correlation between TyG-BMI as a surrogate marker of IR and hypertension in the American population is still unclear. TyG-BMI is a composite index composed of fasting blood tests [triglycerides (TG), fasting blood glucose (FPG)] and body mass index (BMI), which can identify early IR risk and have broad clinical value^16^.

Although the high correlation between IR and HTN has been widely recognized, traditional IR evaluation methods are complex and not suitable for large-scale clinical investigations. Some studies have shown that TyG-BMI can be used as a substitute indicator for IR, but the relationship between TyG-BMI and HTN is not yet fully studied. Therefore, this study aims to validate the correlation between TyG-BMI and HTN in the American population using data from the NHANES database, providing a basis for the clinical application of TyG-BMI.

## Materials and methods

### Study design and population

The National Health and Nutrition Examination Survey (NHANES) is a nationally representative study conducted by the National Center for Health Statistics (NCHS) to assess the health and nutritional status of Americans. This research was performed by trained healthcare professionals. They investigated and collected data on demographics, diet, examinations, and laboratory findings to estimate the prevalence of major diseases and their associated risk factors. For specific information about the database, please visit the NHANES official website at https://www.cdc.gov/nchs/nhanes/. The NHANES study has obtained approval from the Ethics Review Committee of the National Health and Nutrition Examination Survey (NHCS), and all participants have filled out the written informed consent form. We selected NHANES data from 2017 to 2020 to analyze the association between the TyG-BMI and HTN. Exclusion criteria were (1) individuals aged < 20 years, (2) missing any of the metrics used to calculate the TyG-BMI [TG, FPG, height, weight), and (3) individuals with incomplete information on covariates.

### Measurement of the TyG-BMI

The TyG-BMI was the exposure factor and was calculated by the following equation^16^: TyG-BMI = TyG × BMI, where TyG = Ln[TG (mg/dL) × FPG (mg/dL)/2]^17^, and BMI = weight (kg)/height (m^2^). TG was determined using the Wahlefeld method, and FPG was determined by enzymatic methods.

### Definition of HTN

We determined whether participants have HTN based on their answers to the questionnaire, “Has your doctor told you that you have high blood pressure?” If the participants answered “yes” they were included in the group with HTN. The NHANES database diagnoses whether a patient has hypertension based on “The Seventh Report of the Joint National Committee on Prevention, Detection, Evaluation, and Treatment of High Blood Pressure: the JNC 7 report”^18^.

### Measurement of covariates

Covariates include demographic information [age, race, education level, marital status, ratio of family income to poverty (PIR)], body measurement indicators [waist circumference (WC)], smoking status, drinking status, physical activity (PA), diabetes, and laboratory measurement indicators include total cholesterol (TC), High-density lipoprotein cholesterol (HDL-C), Low-density lipoprotein cholesterol (LDL-C). According to the smoking status of participants, they are classified as “current smokers”, “former smokers”, or “never smokers”. According to the Substance Abuse and Mental Health Services Administration (SAMHSA) guidelines^19^, drinking status was divided into three categories: never drinking (0 g/ day), moderate drinking (< 70 g/ day for men and < 56 g/ day for women), and excessive drinking (≥ 70 g/ day for men and ≥ 56 g/ day for women). According to the 2020 World Health Organization (WHO) Physical Activity and Sedentary Behavior Guidelines^20^, PA is divided into two categories: participants who engage in high-intensity activities for more than 75 min per week or moderate-intensity activities for more than 150 min per week are considered to have an active aerobic PA level, otherwise they are considered inactive. Diabetes status was determined by the participant’s answer to the question, “Has your doctor ever told you that you have diabetes?“, with a “yes” response being included as having diabetes. For a detailed explanation of the methods used to measure laboratory indicators such as blood lipids and glucose, please refer to the Laboratory Data Methods section on the NHANES website. (https://www.cdc.gov/nchs/data/nhanes/nhanes_99_00/lab13_met_lipids.pdf, https://www.cdc.gov/nchs/data/nhanes/nhanes_99_00/lab10am_met_plasma_glucose.pdf)

### Statistical analysis

Continuous variables of a normal distribution were represented by mean ± standard deviation (SD), while skewed distributed variables were represented by interquartile range (IQR). Categorical variables were represented by frequency (%). When comparing baseline characteristics between groups with and without HTN, we use one-way analysis of variance (ANOVA) for normally distributed data, the Kruskal-Wallis H test for skewed distributed data, and chi-square test for categorical variables.

We used a multivariable logistic regression model (odds ratio [OR] and 95% confidence interval [CI]) to evaluate the association between TyG-BMI and HTN. Model 1 adjusted for age, gender, and race, while Model 2 further adjusted for demographic factors (including age, gender, race, education level, marital status, PIR), WC, smoking, alcohol consumption, and physical activity. Model 3 fully adjusted for all covariates. Additionally, we employed a smooth curve fitting to assess the linear relationship between TyG-BMI and HTN. Subgroups were divided by age, gender, race, education level, smoking status, PA, and diabetes, respectively. We analyzed subgroups using multiple logistic regression models and analyzed the significance of interactions between subgroups through likelihood ratio tests. Statistical analysis software included R 3.3.2 (http://www.R-project.org, the R Foundation) and Free Statistics 1.8. A bilateral P-value < 0.05 is considered statistically significant for differences.

## Results

### Study population

**Fig. 1:**
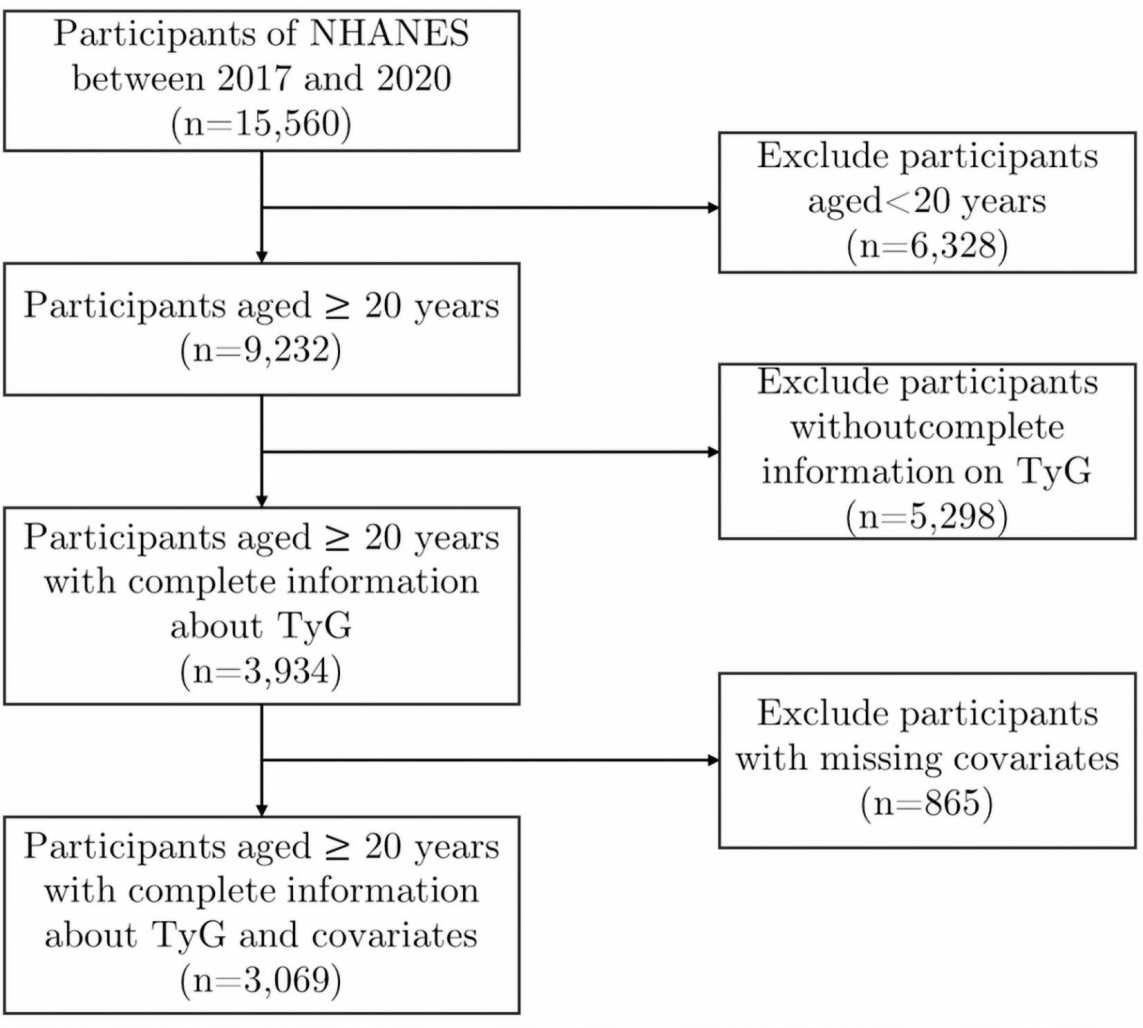
Flowchart of study population screening.

A total of 15,560 participants took part in the NHANES survey during 2017–2020. We excluded 6,328 participants under the age of 20, 5,298 participants who lacked complete information on the calculated TyG-BMI, and 865 individuals with incomplete covariate information. Finally, 3,069 individuals over the age of 20 with complete variable information were included in the study. The crowd screening process diagram is shown in Fig. 1.

### Basic characteristics of the research population

The basic characteristics of the study population are shown in Table 1, the mean age of 3,069 participants was 50.0 ± 17.2 years, of which 48.8% were male. They were categorized into two groups based on whether they had HTN or not. Compared with the non-hypertensive population, those with hypertension had a higher average age (59.0 ± 14.2 vs. 45.2 ± 16.7), higher divorce rate (30.2% vs. 16.6%), and lower level of education (55.9% vs. 61.6%). And the percentage of Non-Hispanic blacks was higher (31.7% vs. 20.4%). In terms of lifestyle habits, the high blood pressure group smoked more (49.7% vs. 39.9%), fewer drinkers (63.7% vs. 73.9%), and fewer regular exercisers (51.5% vs. 62.4%). In terms of disease and physical and chemical indexes, hypertensive population had higher prevalence of BMI, WC, LDL-C, FPG and diabetes, and these differences were statistically significant (*P* < 0.05), while there were no statistically significant differences in gender, PIR, TC, and HDL-C between the two groups (*P* > 0.05).

**Table 1:** Baseline characteristic of participants.

Variables	Total (*n* = 3069)	Non-hypertension (*n* = 1897)	hypertension(*n* = 1172)	*P-*value
Age, years	50.5 ± 17.2	45.2 ± 16.7	59.0 ± 14.2	< 0.001
Gender, n (%)				0.351
Male	1,499 (48.8)	914 (48.2)	585 (49.9)	
Female	1,570 (51.2)	983 (51.8)	587 (50.1)	
Race, n (%)				< 0.001
Mexican American	380 (12.4)	290 (15.3)	90 (7.7)	
Other Hispanics	294 ( 9.6)	182 (9.6)	112 (9.6)	
Non-Hispanic whites	1,117 (36.4)	684 (36.1)	433 (36.9)	
Non-Hispanic black	758 (24.7)	387 (20.4)	371 (31.7)	
Other	520 (16.9)	354 (18.7)	166 (14.2)	
Education level, n (%)				0.007
Below high school	516 (16.8)	300 (15.8)	216 (18.4)	
High school	729 (23.8)	428 (22.6)	301 (25.7)	
Above high school	1,824 (59.4)	1,169 (61.6)	655 (55.9)	
Marital status, n (%)				< 0.001
Married or cohabiting	1,838 (59.9)	1,175 (61.9)	663 (56.6)	
Widow, divorce, or separation	668 (21.8)	314 (16.6)	354 (30.2)	
Unmarried	563 (18.3)	408 (21.5)	155 (13.2)	
PIR	2.7 ± 1.6	2.7 ± 1.6	2.6 ± 1.6	0.054
BMI, (kg/m2)	29.9 ± 7.4	28.8 ± 7.0	31.8 ± 7.7	< 0.001
WC, (cm)	101.0 ± 17.3	97.4 ± 16.6	106.8 ± 16.9	< 0.001
TC, (mg/dl)	183.4 ± 41.3	184.5 ± 40.0	181.7 ± 43.4	0.068
TG, (mg/dl)	89.0 (60.0, 131.0)	83.0 (57.0, 123.0)	99.0 (68.0, 141.0)	< 0.001
LDL-C, (mg/dl)	108.6 ± 36.1	110.4 ± 34.9	105.9 ± 37.8	< 0.001
HDL-C, (mg/dl)	53.8 ± 16.0	54.2 ± 15.7	53.1 ± 16.4	0.069
Smoking, n (%)				< 0.001
Never	1,730 (56.4)	1,141 (60.1)	589 (50.3)	
Current	569 (18.5)	354 (18.7)	215 (18.3)	
Former	770 (25.1)	402 (21.2)	368 (31.4)	
Drinking, n (%)				< 0.001
Never drinking	863 (28.1)	459 (24.2)	404 (34.5)	
Moderate drinking	2,147 (70.0)	1,401 (73.9)	746 (63.7)	
Excessive drinking	59 ( 1.9)	37 (2)	22 (1.9)	
Physical activity, n (%)				< 0.001
Inactive	1,282 (41.8)	714 (37.6)	568 (48.5)	
Active	1,787 (58.2)	1,183 (62.4)	604 (51.5)	
Fasting glucose	112.9 ± 36.6	106.6 ± 29.9	123.2 ± 43.5	< 0.001
Diabetes, n (%)				< 0.001
Non-diabetic	2,595 (84.6)	1,762 (92.9)	833 (71.1)	
Diabetes	474 (15.4)	135 (7.1)	339 (28.9)	
TyG	8.5 ± 0.7	8.4 ± 0.6	8.7 ± 0.7	< 0.001
TyG-BMI	255.7 ± 71.1	242.7 ± 66.9	276.6 ± 72.7	< 0.001

Continuous variables are expressed as mean ± SD or median (IQR), and data for categorical variables are expressed as numbers (%).

PIR, Ratio of family income to poverty; BMI, body mass index; WC, waist circumference; TC, total cholesterol; TG: triglyceride; LDL-C, low-density lipoprotein cholesterol; HDL-C, high-density lipoprotein cholesterol; TyG, triglyceride glucose; TyG-BMI, triglyceride glucose-body mass index.

### Correlation between TyG-BMI and HTN

One-way logistic regression analysis shows that age, race, education level, marital status, BMI, WC, TG, LDL-C, smoking status, drinking status, PA, and diabetes may be related to HTN (Table 2).

**Table 2:** Results of univariate analysis of hypertension.

Variable	OR (95%CI)	*P*-value
Age, years	1.054 (1.049 ~ 1.06)	< 0.001
Gender, n (%)		
Male	Ref	
Female	0.933 (0.807 ~ 1.079)	0.351
Race, n (%)		
Mexican American	Ref	
Other Hispanics	1.983 (1.42 ~ 2.768)	< 0.001
Non-Hispanic whites	2.04 (1.564 ~ 2.66)	< 0.001
Non-Hispanic black	3.089 (2.344 ~ 4.071)	< 0.001
Other	1.511 (1.12 ~ 2.039)	0.007
Education level, n (%)		
Below high school	Ref	
High school	0.977 (0.777 ~ 1.228)	0.84
Above high school	0.778 (0.638 ~ 0.95)	0.014
Marital status, n (%)		
Married or cohabiting	Ref	
Widow, divorce, or separation	1.998 (1.67 ~ 2.39)	< 0.001
Unmarried	0.67 (0.55 ~ 0.83)	< 0.001
PIR	0.957 (0.914 ~ 1.001)	0.054
BMI, (kg/m2)	1.058 (1.047 ~ 1.069)	< 0.001
WC, (cm)	1.034 (1.029 ~ 1.038)	< 0.001
TC, (mg/dl)	0.998 (0.997 ~ 1)	0.069
TG, (mg/dl)	1.003 (1.002 ~ 1.005)	< 0.001
LDL-C, (mg/dl)	0.997 (0.994 ~ 0.999)	0.001
HDL-C, (mg/dl)	0.996 (0.991 ~ 1)	0.069
Smoking, n (%)		
Never	Ref	
Current	1.177 (0.967 ~ 1.432)	0.105
Former	1.773 (1.492 ~ 2.108)	< 0.001
Drinking, n (%)		
Never drink alcohol	Ref	
Drink alcohol	0.605 (0.515 ~ 0.71)	< 0.001
Drink heavily	0.676 (0.392 ~ 1.164)	0.158
Physical activity, n (%)		
Inactive	Ref	
Active	0.642 (0.554 ~ 0.744)	< 0.001
Fasting glucose	1.015 (1.012 ~ 1.017)	< 0.001
Diabetes, n (%)		
Non-diabetic	Ref	
Diabetes	5.312 (4.281 ~ 6.591)	< 0.001
TyG	1.903 (1.696 ~ 2.135)	< 0.001
TyG-BMI	1.007 (1.006 ~ 1.008)	< 0.001

Ref, reference; PIR, Ratio of family income to poverty; BMI, body mass index; WC, waist circumference; TC, total cholesterol; TG: triglyceride; LDL-C, low-density lipoprotein cholesterol; HDL-C, high-density lipoprotein cholesterol; TyG, triglyceride glucose; TyG-BMI, triglyceride glucose-body mass index.

Multifactorial logistic regression analysis found that TyG-BMI was positively associated with HTN after the complete adjustment of covariates. When TyG-BMI was represented as a continuous variable, for every 10-unit increase in the TyG-BMI in the fully adjusted variable (Model 3), the risk of HTN increased by 4.3% (95% CI: 1.007–1.08, *P* = 0.018). When TyG-BMI was used as the Quartile, after full adjustment (model 3), there was still a positive association between the TyG-BMI and the increased risk of HTN. Compared with the smallest Quartile Q1 (≤ 205.44), the fully adjusted OR values of Q2 (205.49–244.86), Q3 (244.88–293.43), and Q4 (≥ 293.57) are 1.442 (95% CI: 1.074–1.935, *P* = 0.015), 2.069 (95% CI: 1.447–2.962, *P* < 0.001), 2.347 (95% CI: 1.427–3.859, *P* < 0.0002), respectively, each model has statistical significance (Table 3).

**Table 3:** Multivariate regression analysis of TyG-BMI and hypertension.

Variable	Unadjusted		Model 1		Model 2		Model 3	
	OR (95%CI)	*P*-value	OR (95%CI)	*P*-value	OR (95%CI)	*P*-value	OR (95%CI)	*P*-value
TyG-BMI, per 10 U	1.072 (1.06 ~ 1.083)	< 0.001	1.085 (1.071 ~ 1.099)	< 0.001	1.06 (1.029 ~ 1.091)	< 0.001	1.043 (1.007 ~ 1.08)	0.0176
Q1 (≤ 205.44)	1(Ref)		1(Ref)		1(Ref)		1(Ref)	
Q2 (205.49–244.86)	2.033 (1.618 ~ 2.555)	< 0.001	1.655 (1.286 ~ 2.131)	< 0.001	1.426 (1.079 ~ 1.884)	0.0125	1.442 (1.074 ~ 1.935)	0.0149
Q3 (244.88–293.43)	3.029 (2.419 ~ 3.792)	< 0.001	2.861 (2.228 ~ 3.673)	< 0.001	2.108 (1.529 ~ 2.908)	< 0.001	2.069 (1.446 ~ 2.962)	< 0.001
Q4 (≥ 293.57)	4.091 (3.27 ~ 5.119)	< 0.001	4.651 (3.614 ~ 5.985)	< 0.001	2.586 (1.671 ~ 4.001)	< 0.001	2.347 (1.427 ~ 3.859)	< 0.002
P for trend		< 0.001		< 0.001		< 0.001		< 0.003

Q1, Q2, Q3, Q4: Quartiles of TyG-BMI.

Model 1 adjust for Age, Gender, Race.

Model 2 adjust for Model 1 + Education level, Marital status, PIR, WC, Smoking status, Drinking, Physical activity.

Model 3 adjust for Model 1 + Model 2 + TC, LDL-C, HDL-C.

Ref, reference; PIR, ratio of family income to poverty; WC, waist circumference; TC, total cholesterol; LDL-C, low density lipoprotein cholesterol; HDL-C, high-density lipoprotein cholesterol; TyG-BMI, triglyceride glucose-body mass index.

**Fig. 2:**
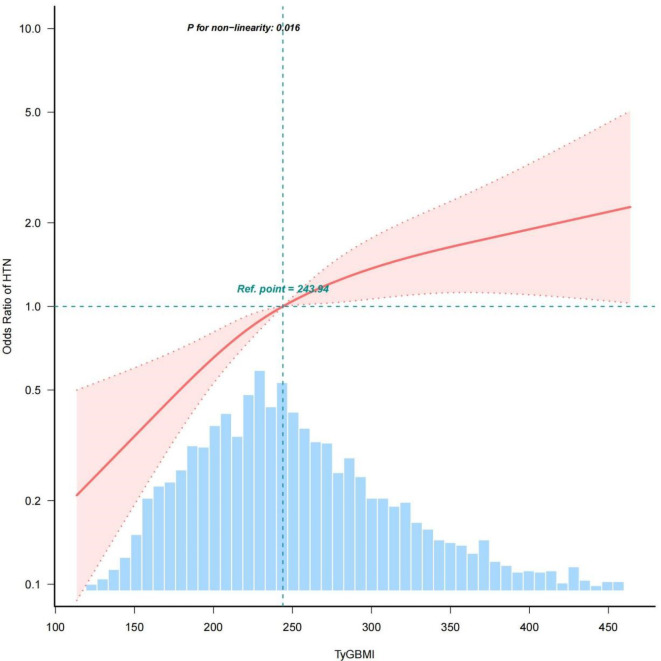
Relationship between TyG-BMI and HTN. The solid red line representing the predicted value and the dashed area representing the 95% confidence interval.

In addition, the smoothed curve fitting analysis (Fig. 2) showed a nonlinear relationship between TyG-BMI and HTN (*P* = 0.016) after adjustment for all covariates(including age, race, education level, marital status, the ratio of family income to poverty, waist circumference, smoking, drinking, physical activity, diabetes, and TC, HDL-C, LDL-C). We further conducted an inflection point analysis on the curve (Table 4) and found that when the TyG-BMI is less than 242.192, every 1 increase in TyG-BMI value increases the risk of HTN by 1.1%. When the TyG-BMI was greater than 242.192, the increase in the risk of HTN with increasing TyG-BMI was not statistically significant (*P* > 0.05).

**Table 4:** Analysis of inflection points.

TyG-BMI	Adjusted Model	
	OR (95%CI)	*P*-value
< 242.192	1.011 (1.001 ~ 1.021)	0.035
≥ 242.192	1.003 (0.999 ~ 1.008)	0.1253
Likelihood Ratio test		0.028

### Subgroup analysis

**Fig. 3:**
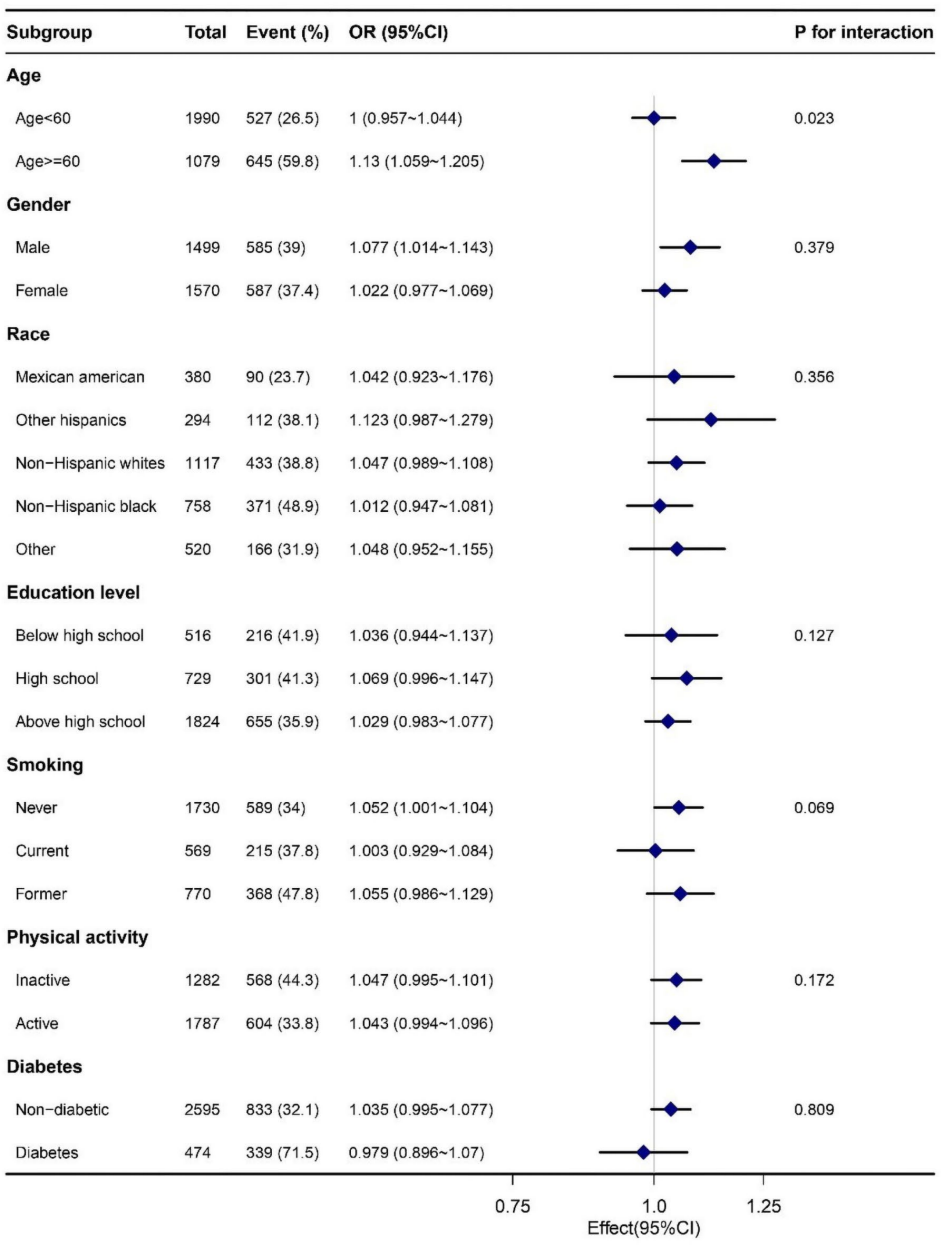
The forest plot of the subgroup analysis of TyG-BMI and HTN.

To test the robustness of the results, we conducted multiple logistic regression analyses after stratification by age, gender, race, education level, smoking status, physical activity, and diabetes status. After fully adjusting for covariates other than stratified variables, we found that the positive association between TyG-BMI and HTN remained stable among all subgroups except the age group. After age stratification, significant interactions were observed (P for interaction < 0.05), indicating that the correlation between TyG-BMI and HTN was more significant when the age was greater than 60 years old (Fig. 3).

## Discussion

Through cross-sectional analysis of 3,069 adults in the NHANES database from 2017 to 2020, we found that the TyG-BMI is positively correlated with the presence of HTN with self-reported diagnosis, and the relationship remains robust after fully adjusted (Table 3). We explored the inflection point for the association of TyG-BMI with hypertension. However, the clinical significance and reliability of this critical value need further verification (Fig. 2). The results of subgroup analysis showed significant interaction after age stratification, and there is no significant difference in the correlation between TyG-BMI and HTN risk among other subgroups (gender, race, education level, smoking status, physical activity, and diabetes status).

Our finding suggested a significant correlation between TyG-BMI and the incidence of HTN in adults over 20 years in the United States, which is consistent with previous findings in Asian populations such as China, Japan, and Thailand^20–22^. Our finding suggests that TyG-BMI, as an easily accessible indicator, has a stable association with the occurrence of HTN in different ethnic groups. Although studies in both Asian and American populations have found a positive correlation between TyG-BMI and the prevalence of HTN, the significance of the association at different ages is somewhat controversial. A sub-analysis of a cross-sectional study on Chinese adults showed no significant difference in TyG-BMI among different age subgroups^24^. Another subgroup analysis of East Asian populations, including China and Japan, found that TyG-BMI was more significantly correlated with HTN in middle-aged adults^25^, whereas a subgroup analysis of a Japanese population with normal blood glucose found that the association was more pronounced in young individuals^21^. Our subgroup analysis showed a more significant association between TyG-BMI and HTN in the US population aged 60 years or older, probably due to cellular senescence, mitochondrial dysfunction, susceptibility to metabolic disorders and weakened self-regulatory ability in the elderly^26,27^, so that older adults with high TyG-BMI are more susceptible to abnormalities of glucose-lipid metabolism and induced HTN. The controversy surrounding the association between age and TyG BMI and hypertension in different study populations may be related to dietary and lifestyle habits in different regions, and further research can be conducted on this issue in the future.

The mechanism of TyG-BMI and HTN association remains unclear. Studies have shown that HTN is strongly related to impaired fasting glucose^28^and abnormal glucose tolerance^29^, and most patients with HTN are accompanied by metabolic system disorders^30^, among which IR and obesity are closely related to increased risk of blood pressure^31,32^. IR promotes abnormal activation of the Renin-Angiotensin-Aldosterone System (RAAS) and the Sympathetic Nervous System (SNS). Increased secretion of Ang II and aldosterone leads to constriction of small arteries throughout the body and increased vascular resistance, which promotes reabsorption of water and sodium by renal tubules, leading to an increase in blood volume and elevated blood pressure^33^. In addition, IR down-regulates nitric oxide synthesis, exacerbates vascular and systemic inflammation, and leads to vascular endothelial dysfunction, which also induces HTN^34-36^. What’s more, previous studies have shown a significant interaction between BMI, one of the commonly used indicators of obesity, and the risk of HTN^37^^38^,. Obesity not only exacerbates insulin resistance, but also produces reactive oxygen species (ROS) and a variety of inflammatory factors, including IL-1 and IL-6, which aggravate vascular damage and endothelial dysfunction, ultimately leading to HTN^39^. Therefore, monitoring TyG-BMI may be a clue to the presence of hypertension.

This study examined whether there is a correlation between TyG-BMI and HTN in the US population, having a diverse population base, and adjusting for major lifestyle factors. However, this study also has certain limitations. Firstly, cross-sectional studies can only analyze associations and cannot establish causal inferences. Secondly, we cannot exclude the residual confounding effects of unmeasured and unknown factors. Additionally, since blood pressure values on different days were not measured in the database, we did not use blood pressure values as the basis for diagnosing HTN. Instead, we used the doctor’s diagnosis results to determine whether patients had HTN, which may have led to bias in some hypertensive patients who did not seek medical treatment. We cannot determine whether patients have been diagnosed with hypertension based on their real-time blood pressure measurements, nor can we ascertain their treatment status or the effectiveness of their blood pressure control. Therefore, we are unable to exclude the potential impact of treatment on various measured indicators, and we cannot conduct subgroup analyses based on blood pressure categories. This limitation may introduce certain biases into our study. Therefore, more rigorous prospective cohort studies or randomized controlled trials are needed in the future to determine the causal relationship between the TyG-BMI and HTN and minimize the interference of confounding factors to further validate the reliability of the results of this study.

## Conclusion

In conclusion, our finding suggested that TyG-BMI is positively associated with the risk of HTN in Americans aged 20 years or older, and this association is more pronounced in those aged 60 years or older. Thus, early monitoring of TyG-BMI may reduce the risk of HTN in the elderly population, thereby reducing the global healthcare burden.

## Electronic supplementary material

Below is the link to the electronic supplementary material.

Supplementary Material 1

## Data Availability

The data analyzed in this study came from publicly available databases and can be obtained from the following link: https://www.cdc.gov/nchs/nhanes/.

## Abbreviations

TyG-BMI: Triglyceride glucose-body mass index
IR: insulin resistance
Ang II: Angiotensin II
WHtR: Waist-to-height ratio
HTN: hypertension
OR: Odds ratio
BMI: Body mass index
VAI: Visceral adiposity index
WC: Waist circumference
LAP: Lipid accumulation product index
NHANES: The National Health and Nutrition Examination Survey
OR: Odds ratio
HIEC: high insulin-normal glucose clamp
HOMA-IR: Homeostasis model assessment
TyG: triglyceride glucose
TyG-WC: Triglyceride glucose-Waist Circumference
TG: triglyceride
FPG: fasting plasma glucose
BMI: body mass index
NHANES: The National Health and Nutrition Examination Survey
NCHS: National Center for Health Statistics
PIR: Ratio of family income to poverty
TC: Total cholesterol
LDL-C: Low-density lipoprotein cholesterol
HDL-C: high-density lipoprotein cholesterol
