# Supplementary material for: Association between triglyceride glucose combined with body mass index and hypertension in the NHANES 2017 to 2020
Source: Sci Rep. 2025 Mar 17;15:9092. doi: 10.1038/s41598-025-93723-w (PMC11914623; doi:10.1038/s41598-025-93723-w)
Supplement: Supplementary file 1 — Supplementary Material 1 [file 41598_2025_93723_MOESM1_ESM.docx]

| **Variable** |  | **Model 1** | | |  | **Model 2** | |  | **Model 3** | |
| --- | --- | --- | --- | --- | --- | --- | --- | --- | --- | --- |
|  |  | **OR (95%CI)** | | **P** |  | **OR (95%CI)** | **P** |  | **OR (95%CI)** | **P** |
| TyG-BMI  per 10 U |  | 1.046 (1.012~1.083) | =0.008 | |  | 1.046 (1.013~1.080) | =0.005 |  | 1.086  (1.073~1.102) | ＜0.001 |

Supplementary Material Table 1 Sensitivity analysis

Model 1 Adjust for Age, Gender, Education level, Marital status, PIR, WC, Smoking status, Drinking, Physical activity, TC, LDL-C, HDL-C

Model 2 Adjust for Age, Race, Education level, Marital status, PIR, WC, Smoking status, Drinking, Physical activity, TC, LDL-C, HDL-C

Model 3 Adjust for Gender, Race, Education level, Marital status, PIR, WC, Smoking status, Drinking, Physical activity, TC, LDL-C, HDL-C

Supplementary Material Table 2 Multivariate regression analysis of TyG-BMI and hypertension

Adjust for Gender, Age, TyG-BMI

| **Variable** |  | **OR (95%CI)** | **P-value** | |
| --- | --- | --- | --- | --- |
|  |  |  |  |  |
| Drink heavily |  | 2.057  (1.150~3.659) | =0.014 |  |

| **Variables** | **N=9232** | **N=3096** | **p value** |
| --- | --- | --- | --- |
| Age, years | 50.41±17.196 | 50.5 ± 17.2 | =0.01 |
| Gender, n (%) |  |  | 0.578 |
| Male | 4479 (48.5) | 1,499 (48.8) |  |
| Female | 4753 (51.5) | 1,570 (51.2) |  |

Supplementary Material Table 3 Sample consistency test
